# Autologous adipose-derived stromal cell treatment for patients with refractory angina (MyStromalCell Trial): 3-years follow-up results

**DOI:** 10.1186/s12967-019-2110-1

**Published:** 2019-11-12

**Authors:** Abbas Ali Qayyum, Anders Bruun Mathiasen, Steffen Helqvist, Erik Jørgensen, Mandana Haack-Sørensen, Annette Ekblond, Jens Kastrup

**Affiliations:** 1 Department of Cardiology & Cardiac Catheterization Laboratory 2014, Rigshospitalet University of Copenhagen, Inge Lehmanns Vej 7, 2100 Copenhagen, Denmark; 2 Department of Cardiology, Gentofte University of Copenhagen, Kildegårdsvej 28, 2900 Hellerup, Denmark; 3 Cardiology Stem Cell Centre 9302, Rigshospitalet University of Copenhagen, Henrik Harpestrengsvej 4C, 2100 Copenhagen, Denmark

**Keywords:** Adipose derived stromal cells, Chronic ischemic heart disease, Regeneration, Stem cell therapy, Refractory angina

## Abstract

**Background:**

Stem cell therapy is investigated as a treatment option for patients with ischemic heart disease. In this study, long-term safety and efficacy of autologous intra-myocardial injections of adipose-derived stromal cells (ASCs) was studied in patients with refractory angina.

**Methods:**

Sixty patients with coronary artery stenosis and preserved left ventricular ejection fraction were 2:1 randomised to intramyocardial injections of ASCs or saline and followed for 3 years.

**Results:**

For patients in the ASC group, the bicycle exercise time and the exercise performance in watt were un-changed (383 ± 30 s to 370 ± 44 s, P = 0.052 and 81 ± 6 to 78 ± 10, P = 0.123, respectively), but the performance in METs was reduced significantly (4.2 ± 0.3 to 4.0 ± 0.4, P = 0.027) during the follow-up period. However, in the same period, there was in the placebo group a significant decline in bicycle exercise time (437 ± 53 s to 383 ± 58 s, P = 0.001), the exercise performance measured in watt (87 ± 12 W to 80 ± 12 W, P = 0.019) and in METs (4.5 ± 0.4 to 4.1 ± 0.4, P = 0.002). Moreover, angina measured as CCS class was significantly reduced in the ASC group but not in the placebo group (2.5 ± 0.9 to 1.8 ± 1.2, P = 0.002 and 2.5 ± 0.8 to 2.1 ± 1.3, P = 0.186, respectively). However, no significant change was observed between the two groups.

**Conclusions:**

Patients receiving ASCs had improved cardiac symptoms and unchanged exercise capacity, in opposition to deterioration in the placebo group.

*Trial registration* ClinicalTrials.gov Identifier: NCT01449032*. Registered 7 October 2011*—*Retrospectively registered*, https://www.clinicaltrials.gov/ct2/show/NCT01449032?term=jens+kastrup&rank=7

## Background

One of the leading reasons for death worldwide is related to ischemic heart disease [1]. Improvements in medical and interventional therapies have made the disease a chronic illness for a lot of patients [2]. Even though, the patients are not suffering from cardiac symptoms, the disease is in progress and it is a matter of time before patients develop cardiac symptoms and cardiac events. Atherosclerosis in coronary arteries initially results in asymptomatic left ventricle dysfunction, which reduces cardiac output leading to left ventricle overload and dilatation, and over time it leads to symptomatic left ventricular dysfunction due to ischemia, apoptosis or necrosis. The symptoms related to ischemic myocardium can be reduced using anti-anginal medical or invasive therapies but the underlying disease does not disappear. Stem cell therapy can potentially regenerate ischemic myocardium and is investigated for that purpose in clinical trials. One of the promising cell sources is adipose-derived stromal cells (ASCs), which can be obtained easily from adipose tissue on the abdomen and is comparable to the bone marrow derived mesenchymal stromal cells (MSCs) [3–7].

Previously, a few studies have used autologous adipose derived cells for patients with ischemic heart disease, but these cells were freshly harvested non-culture expanded cells [8–10]. Thus, a limited non-homogenous cell population was given to each patient. Ex vivo culture expansion of ASCs gives the opportunity to deliver a more homogenous and a larger amount of autologous cells to each patient. The anti-apoptotic effect and angiogenesis mediated by ASCs have preclinical been suggested to be related to cytokines such as IGF-I and VEGF [11, 12]. In a porcine model of acute myocardial infarction, intracoronary administration of freshly harvested ASCs showed a significant reduced size of myocardial perfusion defect compared to the control group [13].

We assessed the safety and efficacy of intra-myocardial delivered vascular endothelial growth factor (VEGF-A_165_) stimulated culture expanded ASCs in patients with chronic ischemic heart disease (CIHD) and refractory angina in a double-blinded placebo-controlled clinical study (MyStromalCell trial) [14]. After 6 months, ASC treatment was considered safe and the primary endpoint bicycle exercise capacity increased significantly in the ASC treated group which was not the case for patients who received intramyocardial injections of saline [15].

With this, the final 3 years follow-up results from MyStromalCell trial will be presented. These results are the first long-term data on intramyocardial delivered ASCs in patients with CIHD and refractory angina.

## Methods

### Study overview

MyStromalCell trial is a single-center phase II, double-blinded, placebo-controlled study investigating the safety and efficacy of intra-myocardial injections of autologous ASCs in patients with CIHD and refractory angina.

The study protocol was approved by the Danish National Ethical Committee (02-268856) and Danish Medicines Agency (2612–2867), and complied with the Declaration of Helsinki. The study is registered in ClinicalTrials.gov (NCT01449032). The Good Clinical Practice Unit of the Capital Region, Denmark monitored the trial throughout the 3 years study period. All patients received oral and written information about the study and signed a written informed consent prior to inclusion.

### Patient population

In total, 60 patients were enrolled aged 30–80 years old with left ventricular ejection fraction (LVEF) > 40% and ≥ 1 significantly coronary artery stenosis not amenable for revascularization. The decision was made by the Heart team, which consisted of study unrelated persons. Despite optimal anti-anginal therapy, the patients had significant heart symptoms.

Inclusion and exclusion criteria are described in Table 1. The patients were randomized 2:1 to ASC or saline injections.

**Table 1:** Inclusion and exclusion criteria

*Inclusion criteria*
Age between 30 and 80 years
Moderate to severe angina (CCS Angina Class II–III) or angina equivalent dyspnea (NYHA class II–III) despite optimal medical therapy
Must have, within 12 months prior to entry, documented coronary angiographic evidence of significant vessel disease, and at least one remaining larger coronary vessel from which new collaterals/vessels could be supplied
Must not be eligible for any other re-vascularization procedures
Left ventricular ejection fraction > 40% measured by echocardiography, SPECT, CT-scan, or MRI
Duration of bicycle ergometry exercise tolerance tests: 2 to 10 min
CABG or PCI within 6 months of entry must have angiography performed at least 4 months after the previous intervention to rule out early restenosis and to document remaining significant vessel disease
Ventricular wall thickness of the treatment zone > 7 mm measured by echocardiography, CT-scan, or MRI
*Exclusion criteria*
Pregnant or lactating women
Clinically significant anemia, leukopenia, leukocytosis, or thrombocytopenia
Conditions other than angina that will limit exercise test (e.g. severe peripheral vascular disease, COPD; FEV_1_ < 1)
Immunocompromised status or currently receiving immunosuppressive therapy
Valvular heart disease requiring surgical intervention
Less than 6 weeks prior to screening: ACS with increase in CK-MB or Troponins/PCI/CABG/Stroke or TIA
History of malignancy < 5 years (except cured non-melanoma skin cancer) or suspicion of current malignancy
Other experimental medications within the last 4 weeks prior to the baseline ETT

*CABG* coronary artery bypass grafting, *CCS* Canadian Cardiovascular Society, *CT* computed tomography, *MRI* magnetic resonance imaging, *NYHA* New York Heart Association, *PCI* percutaneous coronary intervention, *SPECT* single-photon emission computed tomography, *ACS* acute coronary syndrome, *COPD* chronic obstructive pulmonary disease, *ETT* exercise tolerance test, *FEV*_*1*_ forced expiratory volume in 1 s, *TIA* transient ischemic attack

**Fig. 1:**
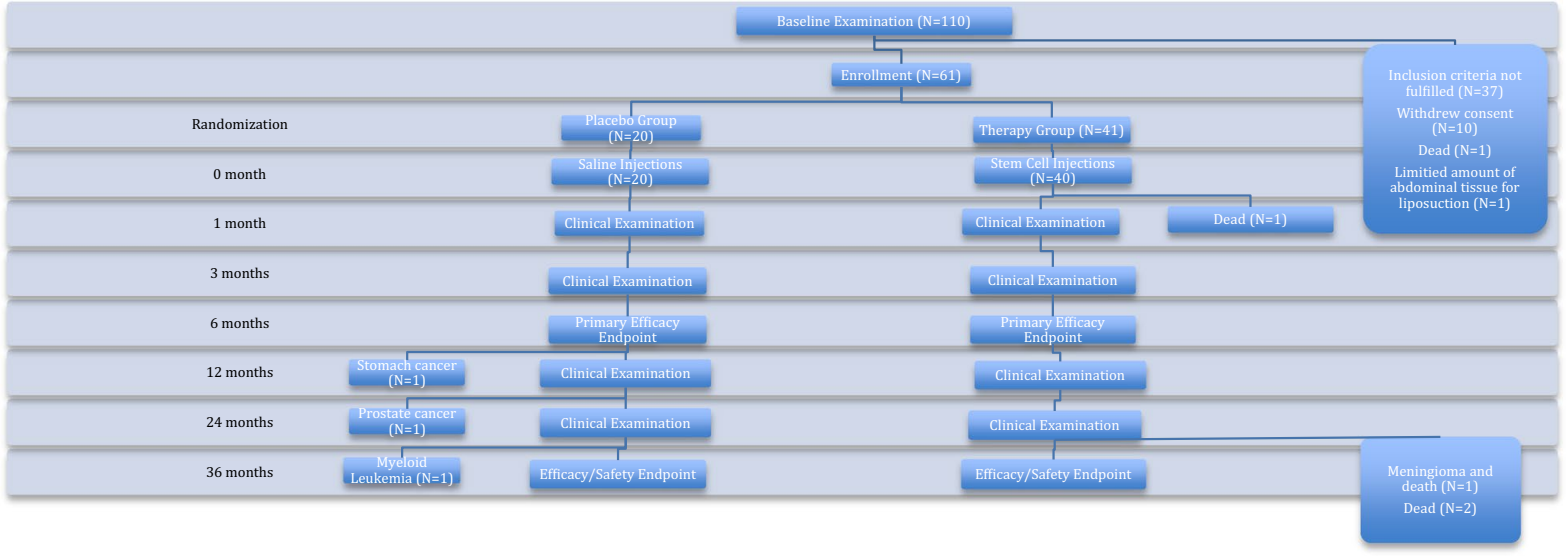
Study design

After intramyocardial delivery of saline or ASCs, the patients were followed 1, 3, 6, 12, 24 and 36 months after the injections (Fig. 1).

### Cell preparation and culturing

In local anesthesia, approximately 100 ml of subcutaneous adipose tissue was obtained from the abdomen of each patient. Isolation and expansion of ASCs has been published previously [14, 15]. Briefly, the lipo-aspirate was washed with phosphate-buffered saline and the adipose tissue was digested by collagenase NB6, neutralized with complete medium containing Dulbecco’s Modified Eagle’s Medium low glucose 1 g/l, 1% Penicillin/Streptomycin (10,000 U/ml and 10.000 μg/ml, respectively) and 10% Fetal Bovine Serum (FBS). Then the suspension was filtered, centrifuged, and re-suspended in complete medium [16]. The isolated stromal vascular fraction was seeded in 75T culture flasks. Every 3–4 days, the complete medium was changed, and the culture was passaged at 80–90% confluence. The ASCs were cultured in serum reduced VEGF-A_165_ medium for 7 days prior to the intramyocardial injections. Each patient was treated with the total amount of cells reached after 2 passages.

### Electromechanical mapping and intramyocardial injection of ASCs

A 3D electromechanical map of left ventricle was created using NOGA^®^ system (Biologics Delivery Systems, CA, US). An 8-french NOGA Myostar^®^ injection catheter was used to deliver 10–15 injections of 0.2 ml of ASCs or saline into the ischemic region of the left ventricle.

### Endpoints

Along with clinical examination, registration of side effects, the patients performed bicycle exercise test at baseline, 3, 6, 12, 24 and 36 months after injections. The efficacy endpoints included changes in Canadian Cardiovascular Society (CCS) and New York Heart Association (NYHA) classification, Seattle Angina Questionnaire, weekly use of nitroglycerin and weekly frequency of angina attacks. Moreover, 1 month after ASC/saline injections, the patients were clinically examined without performing bicycle exercise test.

### Statistical analysis

All statistical analysis was performed using SPSS version 23.0 (SPSS Inc., Chicago, Illinois). Continuous variables are presented as mean ± standard deviation (SD) or 95% confidence interval and categorical variables are presented as numbers and percentages. Paired *t*-test is used for comparison of continuous data within groups while unpaired *t*-test is used for comparison between groups. Repeated measure with autoregressive covariance structure is used for follow-up data with more than two time-points (bicycle exercise data, symptoms, angina attacks, use of short-term nitroglycerin and Seattle Angina Questionnaire). The data were analyzed as intention-to-treat analysis with missing data filled in with last observation carried forward. Categorical data were compared using Fisher’s exact or Chi-square test. A two-sided P-value of < 0.05 was considered statistically significant.

## Results

Forty patients were randomly allocated to the ASC group and treated with 72 ± 45 × 10^6^ ASCs culture expanded for 32 ± 14 days. The injection volume with the expanded ASCs was 3 ml. The cell viability was 89 ± 5%. No signs of contamination with bacteria, yeast or mycoplasma were detected. Remaining 20 patients received intramyocardial saline injections. Baseline characteristics and medicine are shown in Table 2 and 3, respectively.

**Table 2:** Baseline characteristics

Parameter	Placebo (n = 20)	ASC (n = 40)	P-value
Age (years)	65.3 ± 8.7	65.5 ± 9.7	0.94
Male gender	20 (100)	35 (87.5)	0.02
BMI (kg/m^2^)	30.0 ± 4.8	30.0 ± 4.1	0.92
Smoking			0.19
Current	3 (15)	8 (20)	
Previous	16 (80)	23 (57.5)	
Never	1 (5)	9 (22.5)	
Diabetes mellitus	6 (30)	16 (40)	0.57
Hypertension	12 (60)	33 (82.5)	0.06
Previous AMI	10 (50)	26 (65)	0.26
Previous CABG	20 (100)	33 (82.5)	0.08
Previous PCI	15 (75)	28 (70)	0.69
LVEF (%)	54 ± 8	52 ± 8	0.38

*AMI* acute myocardial infarction, *ASC* adipose-derived stromal cell, *BMI* body mass index, *CABG* coronary artery bypass grafting, *LVEF* left ventricular ejection fraction, *n* number of patients, *PCI* percutaneous coronary intervention

**Table 3:** Baseline medication

Medication	Placebo (n = 20)	ASC (n = 40)	P-value
Acetylsalicylic acid	19 (95)	35 (87.5)	0.65
Clopidogrel	7 (35)	11 (27.5)	0.56
ACE-I or ARB	13 (65)	29 (72.5)	0.42
β-blocker	16 (80)	33 (82.5)	1.00
Calcium antagonist	12 (60)	19 (47.5)	0.42
Diuretics	12 (60)	27 (67.5)	0.58
Statins	20 (100)	40 (100)	1.00
Nitrate	19 (95)	28 (70)	0.04
Nicorandil	6 (30)	5 (12.5)	0.16
Ivabradine	2 (10)	4 (10)	1.00

*ACE*-*I* angiotensin-converting enzyme inhibitor, *ARB* angiotensin II receptor blockers, *ASC* adipose-derived stromal cell, *n* number of patients

### Safety

During the 3 years follow-up, 4 patients died. Death of three patients was attributed to a cardiovascular event while 1 of the patient died due to meningioma. One patient died prior to 1-month examination, the other patients died 34, 35 and 36 months after treatment, respectively.

During follow-up time, 4 patients developed cancer. One patient from ASC group developed meningioma. The patients in the placebo group developed prostate cancer, stomach cancer and myeloid leukemia (Table 4).

**Table 4:** Serious adverse events during 3 years follow-up in the placebo and adipose-derived stromal cell (ASC) group

Serious adverse events	Placebo (n = 20)	ASC (n = 40)	P-value
Death	0 (0)	4 (10)	0.291
Hospitalizations			
Myocardial infarction	5 (25)	8 (20)	0.744
Dyspnea	0 (0)	1 (2.5)	1.000
Anemia	2 (10)	3 (7.5)	1.000
Syncope	1 (5)	0 (0)	0.333
Peripheral edema	0 (0)	1 (2.5)	1.000
Angina worsening	12 (60)	14 (35)	0.028
Pneumonia	1 (5)	4 (10)	0.656
PCI	7 (35)	6 (15)	0.101
Heart failure	0 (0)	4 (10)	0.291
TIA/apoplexia cerebri	2 (10)	0 (0)	0.107
Pulmonary embolism	0 (0)	1 (5)	1.000
Cancer	3 (15)	1 (5)	0.103

Values are n (%); P values are calculated using Fischer’s exact test except for angina worsening, which is calculated using Chi-square test

*PCI* percutaneous coronary intervention, *TIA* transient ischemic attack

Six patients did not show up for 36 months follow-up. Four of them were not alive, 1 had stomach cancer and was not able to show up and 1 patient had upper airway infection.

## Exercise tolerance testing

**Fig. 2:**
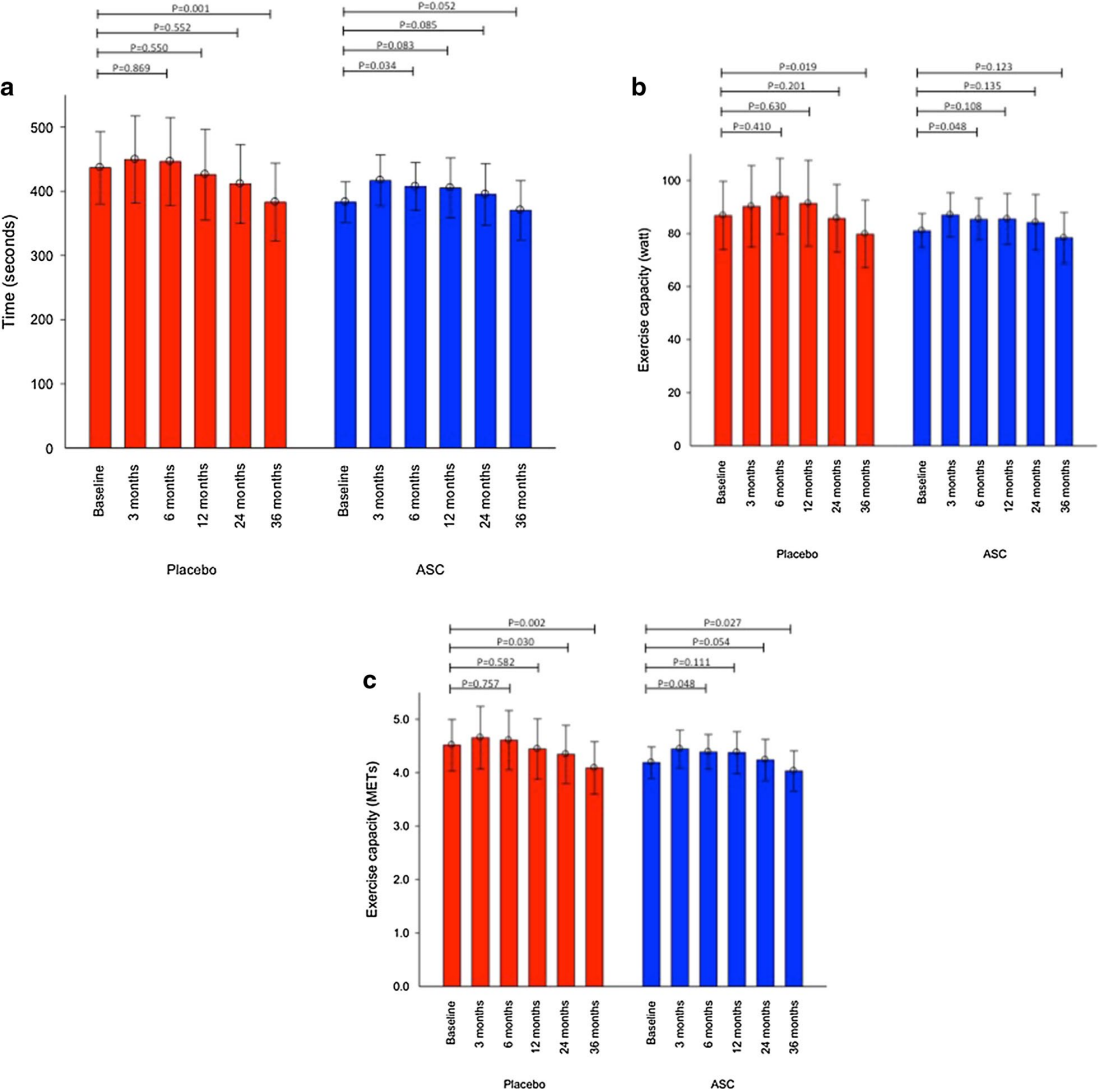
Bicycle exercise test. Performance in **a** time duration, **b** in watt and **c** in METs

At 3 years follow-up, the mean bicycle exercise tolerance time was un-changed in the ASC group from 383 ± 30 s to 370 ± 44 s (P = 0.052), while it decreased significantly in the placebo group from 437 ± 53 s to 383 ± 58 s (P = 0.001) (Fig. 2). A significant decrease was also observed in the placebo group for the performance expressed in watt from 87 ± 12 W at baseline to 80 ± 12 W (P = 0.019), but was un-changed in patients treated with ASCs from baseline to 3 years follow-up (81 ± 6 W to 78 ± 10 W, P = 0.123).

Corresponding metabolic equivalents (METs) was reduced significantly in both the ASC group (from 4.2 ± 0.3 to 4.0 ± 0.4, P = 0.027) and in the placebo group (from 4.5 ± 0.4 to 4.1 ± 0.4, P = 0.002) during the 3 years follow-up period.

There were no significant differences between groups during the 3 years of follow-up (Table 5).

**Table 5:** Bicycle exercise test. Difference between groups

	Follow-up time	Group	Mean difference from baseline	Standard deviation	Mean difference between groups	95% Confidence interval of the difference		P-value
						Lower	Upper	
Time	12 months	Placebo	10	118	34	− 29	97	0.288
		ASC	− 24	113				
	24 months	Placebo	24	98	37	− 25	100	0.239
		ASC	− 13	121				
	36 months	Placebo	51	100	39	− 13	91	0.141
		ASC	12	92				
Watt	12 months	Placebo	− 4.4	24.2	0.4	− 12.3	13.1	0.952
		ASC	− 4.7	22.7				
	24 months	Placebo	1.0	23.0	4.3	− 9.4	18.1	0.529
		ASC	− 3.3	25.9				
	36 months	Placebo	6.6	23.4	4.0	− 7.5	15.5	0.488
		ASC	2.6	19.6				
Mets	12 months	Placebo	0.07	1.00	0.28	− 0.23	0.78	0.277
		ASC	− 0.21	0.88				
	24 months	Placebo	0.17	0.88	0.23	− 0.27	0.74	0.355
		ASC	− 0.07	0.93				
	36 months	Placebo	0.41	0.86	0.27	− 0.16	0.69	0.212
		ASC	0.14	0.72				

### Cardiac symptoms and anti-anginal medication

**Fig. 3:**
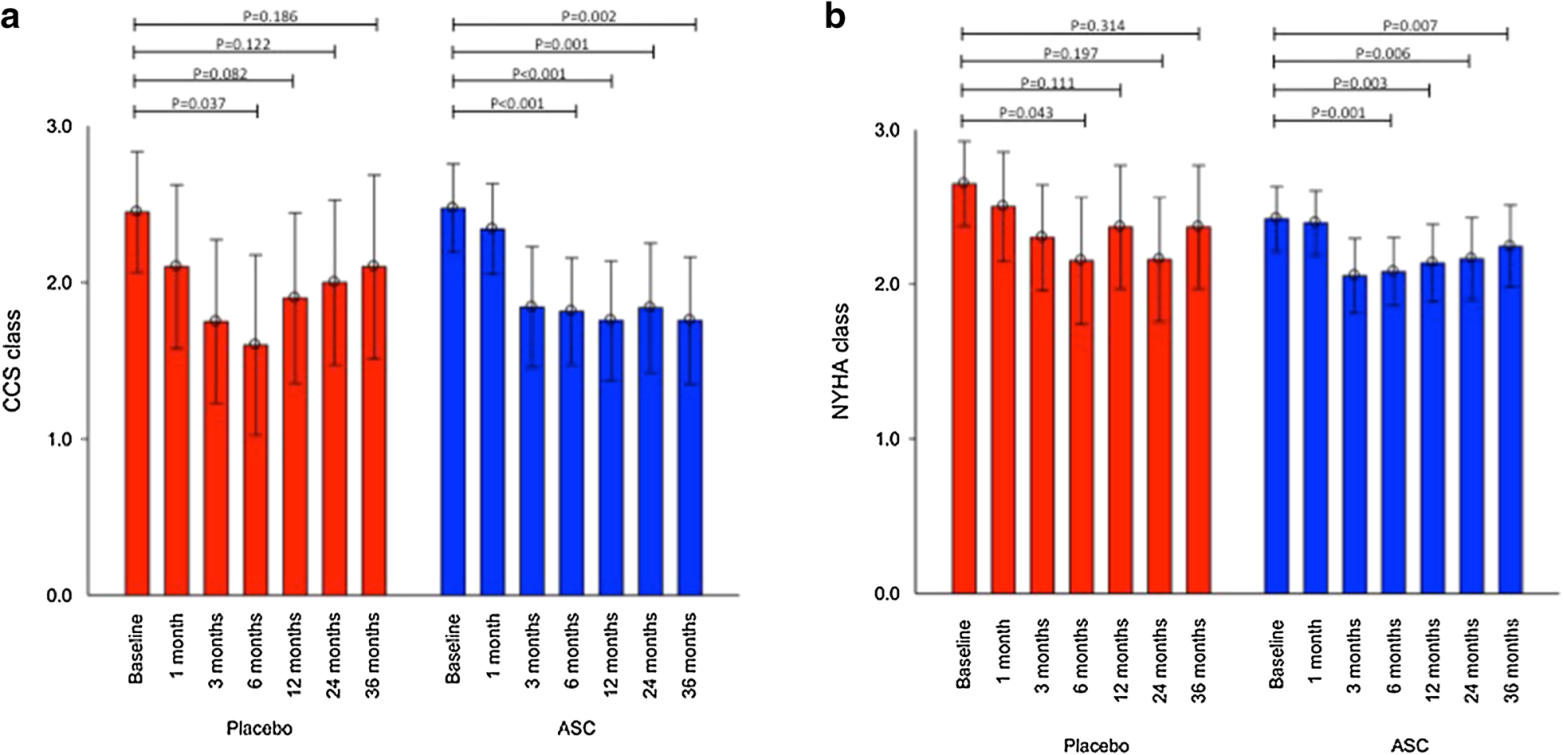
Cardiac symptoms measured as **a** CCS classification and **b** NYHA classification

During the 3 years follow-up period, significant decreased angina was observed measured as CCS classification in the ASC group (2.5 ± 0.9 to 1.8 ± 1.2, P = 0.002) while the symptoms were unchanged the placebo group (2.5 ± 0.8 to 2.1 ± 1.3, P = 0.186) (Fig. 3).

The chest discomfort was significantly reduced in the placebo group during the period from baseline to 6 months follow-up but then increased again while the CCS classification was significantly reduced for patients in ASC group from baseline to 6 months follow-up and remained decreased during the entire follow-up period (Fig. 3).

For NYHA classification, there was a significant reduction observed in the ASC group (2.4 ± 0.6 to 2.2 ± 0.8, P = 0.007) while there was no significant change in the placebo group (2.7 ± 0.6 to 2.4 ± 0.8, P = 0.314).

The patients reported significantly reduced number of weekly angina attacks in the ASC group (P = 0.017) while this number was unchanged in the placebo group (P = 0.425).

In the same period, the use of short-term nitroglycerin was unchanged in both groups (ASC: P = 0.176 and Placebo: P = 0.123).

### Seattle Angina Questionnaire

Significant improved quality-of-life (P < 0.001 and P = 0.045 for ASC and placebo group, respectively), angina stability (P < 0.001 and P = 0.003 for ASC and placebo group, respectively), angina frequency (P < 0.001 and P = 0.012 for ASC and placebo group, respectively) and physical limitation score (P = 0.002 and P = 0.017 for ASC and placebo group, respectively) was observed in both groups but no significant change in overall satisfaction score was seen in any of the groups (P = 0.489 and P = 0.604 for ASC and placebo group, respectively). There were no differences in any parameter between the two groups.

## Discussion

The present study is reporting long-term safety and efficacy data for the first in man randomized, double-blinded placebo-controlled study using VEGF-A_165_ stimulated culture expanded ASCs for patients with refractory angina.

The bicycle exercise performance decreased significantly in the placebo group but was unchanged in the ASC group except for the performance measured in METs at 3 years follow-up.

Cardiac symptoms measured as CCS class and the numbers of weekly angina attacks were significantly reduced during the 3 years of follow-up time for the patients treated with ASCs while there were no changes for the patients receiving saline injections. However, use of short-term nitroglycerin was un-changed in both groups.

For patients in both groups, significant improved quality-of-life, angina stability, angina frequency and physical limitation score was observed.

Chronic ischemic heart disease is a progressive disease. During the follow-up period of 3 years, 13 patients were admitted due to acute myocardial infarction and 26 due to angina worsening. The decrease in bicycle performance in the placebo group illustrates the progressive nature of coronary artery disease.

Interestingly, during the first 6 months follow-up period, significant improvement in heart symptoms of all patients was seen. However, the present long-term data demonstrated that patients allocated into the saline group returned to baseline cardiac symptoms but for patients in the ASC group, the heart symptoms remained decreased during the entire follow-up period. Along with this, the number of weekly angina attacks was only reduced in the ASC group. It can be speculated whether this effect over time in reduced cardiac symptoms would have been seen if only growth factors were injected. However, injection of growth factors may result in rapid dilution while ASCs are bigger in size and may stay in the injected tissue for a time period. Moreover, ASCs may secrete a cocktail of growth factors in a highly complex manner to promote angiogenesis. Nevertheless, for the patients it is highly important that any type of treatment results in decreased morbidity and mortality.

Previous published studies using exercise test as an outcome measure, show similar change in exercise capacity after CD34^+^ injections in patients with chronic ischemic heart disease and refractory angina [17–19]. In these studies, the patients received a standardized dose of cells. In the present study, the patients received the total amount of cells reached after two cell culture expansion passages and thus there were a patient-to-patient variation in the number of cells delivered.

Intra-myocardial injection of freshly harvested adipose-derived cells have been used in patients with refractory angina, which showed that exercise capacity measured as METs in the cell group remained stable while there was a decrease in METs in the placebo group [9]. It is the same tendency as in our study.

Another study also using freshly harvested autologous adipose derived cells injected intramyocardially in patients with ischemic heart failure showed increased maximum oxygen consumption on exercise treadmill in patients receiving cells but did not differed significantly from the placebo group [8].

Another small study delivering freshly harvested adipose-derived cells intracoronary in patients with ST-elevations myocardial infarction showed a trend towards improved LVEF [10].

Previously at our center, we have conducted studies using autologous bone marrow derived mesenchymal stromal cells for patients with chronic ischemic heart disease with and without heart failure [20–22]. These studies have demonstrated improvement in patients self-reported health, increase in LVEF, decreased left ventricular end-systolic volume and reduced amount of scar tissue.

It has been increasingly clear for many research groups, that the expansion of autologous cells in flasks makes it very difficult to deliver a standardized stem cell treatment. New cell culture methods using bioreactor systems to reach a standardized cell count for allogeneic treatments are now available for clinical use [23]. These systems have been used in a phase I trial, to produce an allogeneic cell product (CSCC_ASC) comparable to the one used in this trial as autologous product, for treatment in 10 patients with ischemic heart failure [24]. The aim was to investigate the safety of intra-myocardial injections of 100 million ASCs from healthy donors. No treatment-related side effects were observed.

Furthermore, CSCC_ASC is being tested in a Danish and European multicentre double-blinded placebo-controlled trial for treatment of patients with ischemic heart failure (EudraCT: 2015-001560-19 and EudraCT: 2015-002929-19, respectively) [25, 26].

Moreover, a single-centre study initiated January 2019 is investigating CSCC_ASC in patients with non-ischemic heart failure (EudraCT: 2018-002538-19).

In this study, the total amount of ASCs reached were injected into the patients allocated to the active therapy group, while the adipose tissue obtained from some of the patients in the placebo group, were used for characterization of ASCs [16]. Gene expression levels by quantitative real-time PCR analysis of ASCs showed that transcription of endothelial markers FOXF1, vWF, and VEGFR1 were up-regulated. By flow cytometry, we found the characteristic mesenchymal stem cell markers CD13, CD73, CD90, and CD105 on ASCs and they were lacking HLA-DR, CD19, and CD14.

So, the accumulated experience from clinical stem cell trials, the established safety and efficacy data has led this field to move from autologous to allogeneic therapy, reducing the logistical obstacles meet in previous studies. Furthermore, this field moves on to give a standardized amount of cell therapy safely and equally to all patients. However, as it is known from traditional pharmaceutical therapies, mostly repeated treatment is used. It may be of interest to investigate the role of cell therapy with two treatments within a short period.

The obvious benefit of cell therapy may be that it has the potential to regenerate the ischemic myocardium compared to conventional medical and interventional therapies. However, we did not have long term imaging modalities to detect changes in myocardial perfusion [27]. Moreover, in a selected group of patients the yield of cells and the effect of therapy may be higher [28]. Even though, this study is a randomized clinical trial, there were only male in the placebo group and a trend towards significant difference between the two groups for hypertension and previous coronary artery bypass grafting. Thus, these factors could potential be confounders.

Even though, the primary end-point did not differ between the two groups at 6 months follow-up, there was at 6 months follow-up a significant increase in bicycle exercise capacity in the ASC group, which was not the case for patients in the placebo group.

Probably, a larger amount of patients were needed in this study to detect a significant difference between groups for the primary end point. However, reduced cardiac symptoms are essential for the patient’s quality of life, which was seen only for the patients in the ASC group in this long-term follow-up period.

## Conclusion

In conclusion, patients receiving ASC injections, in this double-blinded placebo-controlled study, had preserved exercise capacity, while patients in the placebo group experienced significantly reduced exercise performance. Additionally, patients receiving ASCs had significantly improved cardiac symptoms, which was not the case for patients allocated in the placebo group.

## Data Availability

The datasets used and/or analyzed during the current study are available from the corresponding author.

## Abbreviations

ASC: adipose-derived stromal cell
CCS: Canadian Cardiovascular Society
CIHD: chronic ischemic heart disease
FBS: Fetal Bovine Serum
LVEF: left ventricular ejection fraction
METS: metabolic equivalents
MSC: mesenchymal stromal cell
NYHA: New York Heart Association
SD: standard deviation
VEGF: vascular endothelial growth factor
